# Switzerland-wide *Neospora caninum* seroprevalence in female cattle and identification of risk factors for infection

**DOI:** 10.3389/fvets.2022.1059697

**Published:** 2022-11-17

**Authors:** Diana S. Gliga, Walter Basso, Flurin Ardüser, Gaia Moore-Jones, Gereon Schares, Patrik Zanolari, Caroline F. Frey

**Affiliations:** ^1^Department of Infectious Diseases and Pathobiology, Vetsuisse Faculty, Institute of Parasitology, University of Bern, Bern, Switzerland; ^2^Clinic for Ruminants, Vetsuisse Faculty, University of Bern, Bern, Switzerland; ^3^Institute of Epidemiology, Friedrich-Loeffler-Institut, Federal Research Institute for Animal Health, Greifswald-Insel Riems, Germany

**Keywords:** *Neospora caninum*, bovine neosporosis, seroprevalence, survey, cattle abortion, risk factor

## Abstract

**Introduction:**

*Neospora caninum* is an important cause of abortion in cattle worldwide. Infection in cattle occurs horizontally by ingestion of oocysts shed by canids or vertically, from an infected dam to the fetus, and may result in abortion, stillbirth, or birth of seropositive offspring. The control of bovine neosporosis is difficult and costly. The objectives of this study were to estimate the current nationwide seroprevalence of *N. caninum* infections in Swiss cattle and to assess risk factors for infection with this parasite.

**Methods:**

We conducted a cross-sectional study with cattle farms randomly selected and stratified according to population size, resulting in a sample of 780 female cattle. The cattle originated from 161 farms distributed over all Switzerland. The serum samples were tested for antibodies against *N. caninum* using a commercial ELISA and if inconclusive, retested using an *in-house* immunoblot technique. To collect farm parameters relevant to *N. caninum* transmission and prevention, farm owners were mailed a questionnaire which addressed topics putatively related to *N. caninum* infection such as husbandry, history of abortion, and presence of dogs on farm. Univariate analysis by generalized linear mixed model (with animal seropositivity as outcome variable) and logistic regression modeling (with farm seropositivity as outcome variable) was conducted on farm parameters investigated in the questionnaire.

**Results:**

By ELISA and immunoblot, 4.2% (33/780) of cattle sera yielded positive results. At the farm level, 16.2% (26/161) of the sampled farms had at least one seropositive animal. The return rate of the valid questionnaires was 54.0%. At the animal level, odds for farm seropositivity were 3.8 times higher when rodents had been recorded by the farmer as a problem on the farm. At the farm-level, two protective factors were identified: rearing of replacement heifers and feeding of concentrated feed.

**Conclusion:**

We recorded a low seroprevalence of *N. caninum* in a random sample of Swiss cattle representative for the years 2017–2018. Based on a questionnaire survey, we could identify risk and protective factors for infection with *N. caninum*, however their biological relevance needs to be confirmed in further studies.

## Introduction

*Neospora caninum* is one of the most important infectious causes of abortion in cattle worldwide. Dogs are definitive hosts and responsible for oocyst shedding. *Neospora caninum* can be maintained also in the sylvatic cycle by wild canids: wolves (1), coyotes (2), and dingoes (3). Interestingly, red foxes are not competent definitive hosts (4). Definitive hosts become infected by ingesting *N. caninum* cysts in placenta (5) or soft tissues (muscle and nervous tissue) of infected intermediate hosts, mainly large ruminants (6). After about 5 days, dogs start to shed oocysts via feces for 2 to 3 weeks (7). These can remain infectious in the environment for months, especially at mild temperatures and in humid conditions (6) until they are ingested by intermediate hosts (horizontal transmission). Bovids are the main natural intermediate hosts of *N. caninum*, but the infection has been also recorded in small ruminants (8), South American camelids (9, 10), cervids (11), pigs (12), birds (13), and small rodents (14, 15). Depending on the source of the primary infection, two modes of vertical transmission have been described in cattle. If the infection is acquired for the first time in a pregnant dam through ingestion of oocysts, it is referred to as exogenous transplacental transmission and it may cause abortion storms in a farm (9, 16). This can be prevented by restricting dog access to pastures, farm buildings or facilities, and feed stores. Once a naïve dam is infected, a chronic process begins in which the parasites migrate to the brain and muscles where they persist as intracellular cysts. Subsequent gestations can trigger parasites recrudescence and rapid replication as tachyzoites which have a tropism for the placenta and fetus This is known as endogenous transplacental transmission (16). The efficiency of transplacental transmission is generally high, and seems to be more efficient in dams with high antibody titers (17). Intrauterine infections can result in abortions or stillbirths, but more often result in the birth of infected but clinically unremarkable cattle that remain infected for life and may abort in the future. *Neospora caninum* positive cattle may experience reproductive failures at any time later due to the reactivation of the infection. This way the parasite can be passed over generations, therefore identification of positive dams is necessary to prevent accumulation of positive cases in the herd. Some studies found a 3–7 times higher risk for abortion in seropositive dairy cattle compared to seronegative [reviewed in (18)], in contrast to beef-cattle that did not show increased abortion rates following an abortion storm (19). In European countries *N. caninum* seroprevalences varied between 16 and 76% for dairy cattle and 41–61% for beef cattle [reviewed in (20)]. Since 2001, neosporosis is a notifiable disease and monitored in Switzerland. According to the ordinance on epizootic diseases [Swiss internal law, 916.401, June 1st 2022, Art. 129 (21)] farmers have the obligation to report abortions in hoofed livestock and in case of frequent occurrence, i.e., more than one abortion over 4 months, the veterinarian must investigate the cause of abortion. Serum, placenta, and fetal material are to be tested for a panel of abortifacient pathogens, but *N. caninum* is not included in this mandatory panel. In 1994, a *N. caninum* seroprevalence of 11.5% was determined in a sample representative of the Swiss cattle population (22). Later on, a case-control study including farms from six Swiss cantons determined a 44% seroprevalence in aborting dams (23). The yearly financial losses due to *N. caninum* in dairy cows were estimated at 9,7 mio Euros per year for Switzerland (24). The same study concluded that the only cost-effective measure to control bovine neosporosis was to discontinue breeding with offspring from seropositive cows. Recent studies in Switzerland investigated the seroprevalence of *N. caninum* infection in small ruminants (i.e., 0.8% in sheep and 0–9% in goats) (8) and in South American camelids (i.e., 3.5% in alpacas and 2.5% in llamas) (9), but data on the current situation of bovine neosporosis in the general cattle population was lacking. The objectives of this study were to estimate the nationwide seroprevalence and distribution of *N. caninum* infections in Swiss cattle and to assess the risk factors for infection with this protozoan parasite in order to gain information on the epidemiology of *N. caninum* infections in cattle in Switzerland.

## Materials and methods

### Sampling

We performed a cross-sectional study with cattle farms randomly selected and stratified according to population size to estimate the nationwide seroprevalence of *N. caninum* infections in Switzerland. A representative set of cattle serum samples was collected from May 2017 through June 2018 within the frame of a previous project (25). A total of 780 female cattle, which derived from 161 randomly selected farms distributed over 25 of the 26 Swiss cantons (one canton, Basel-Stadt, had no farms registered) were sampled. This represented 0.1% (*n* = 1,279,239) of the entire female cattle population in Switzerland at the beginning of the study in 2017 (26). The exact sampling strategy was presented in a previous paper (25). The median of sampled animals per farm was five (2–6 animals per farm). Before visiting farms, official authorization was obtained from the respective Cantonal Veterinary Office. The study was approved by the cantonal committees for animal experimentation of the cantons involved in accordance with the Swiss animal welfare legislation (approval number BE 5/17+). A written consent was obtained from the farmers for enrolling in the study and for the publication of the anonymized results thereof. At sampling, basic data on the farm (owner, farm registration number, and location) and sampled animals (animal identification number, age, sex, breed) were recorded. These animals were clinically unremarkable and abortion status was not inquired at the time of sampling. Normally, blood was collected from the coccygeal vein with a Vacutainer. If not possible, the blood was taken from the jugular vein. Blood samples were cooled for transportation, centrifuged, and the obtained serum was stored at −20°C until analysis.

### Serological analysis

Serum samples were tested for antibodies against *N. caninum* using a commercial ELISA kit for bovines (IDEXX *Neospora caninum* Antibody Test Kit) as indicated by the manufacturer. The sensitivity and specificity of the test were estimated at 100% and 93.3%, respectively (27). For each serum sample, a sample-to-positive ratio (S/P%) was calculated based on the optical density (OD) of the sample and of the positive and negative controls of the kit according to the formula S/P% = (OD sample – OD negative control)/ (OD positive control – OD negative control) × 100. According to the manufacturer, animals with S/P% < 30% were considered negative, inconclusive if 30% ≤ S/P% < 40% and positive if S/P% ≥ 40%. Inconclusive ELISA results were defined using an *in-house* immunoblot technique as previously reported (9) with the exception that the conjugate used was peroxidase-conjugated goat anti-bovine IgG (Jackson ImmunoResearch Europe Ltd, code 101-035-003) diluted 1:600 in PBS-Tween. Immunoblot is the usual confirmatory test in our laboratory due to its high specificity (28, 29). The farm serostatus was considered positive when at least one of the tested animals yielded a seropositive result (animal serostatus).

### Questionnaire

In order to identify possible risk factors for *N. caninum* infections in cattle, a postal questionnaire was sent, after the sampling period ended, to the farms from which the cattle samples originated. The questionnaire included an explicit consent by signature of the participants to participate in the study, and four sections (Supplementary material 1). The first section enquired contact information of the participant and general parameters about their farm: identification number, production type (dairy, beef, or other), herd size (three groups based on 33.3% and 66.6% percentiles of the animal count per farm), and restocking (rearing of replacement heifers, buying-in). In the second section, information related to cattle husbandry was asked for, such as housing system, alpine pasturing, water source, type of feed, proportion of pasture in total feed, feed storage, and history of abortion. In the third section, questions about farm dogs and dog access to the farm (farm premises, or pasture) were asked. The last section asked for information about other animals present in the farm besides cattle and dogs, and about problems with rodents. The questionnaire was translated to French for the farms in the French-speaking part of Switzerland.

### Data analysis

Logistic regression was applied to investigate risk factors at two different levels, farm serostatus and animal serostatus. The farm level model was done using the *glm* function (package “stats”), binomial distribution, logit link function, the binary response variable farm serostatus (“seropositive” or “seronegative”), and as explanatory variable the farm parameters as possible risk factors as inquired in the questionnaire (Supplementary material 1). Some explanatory variables with many categories were pooled according to the biological significance. In total 26 explanatory variables were tested in association with farm serostatus. Multilevel-modeling at animal level was done using function *glmer* (package “lme4” with Laplace method as default approximation), binomial distribution, binary response variable “animal serostatus” with values “seropositive” or “seronegative,” and random factor “farm identification number.” In addition to the 26 explanatory variables described in the previous model (farm parameters), three individual animal parameters were tested: age in years, age group, and breed. Datasets containing the variables cattle and dog population size, density per km^2^, and ratio to 100 inhabitants at municipality level were obtained from Identitas AG (https://www.identitas.ch) and tested as explanatory variables in association with farm serostatus and animal serostatus. Explanatory variables significant at a *p* = 0.05 level in the univariate model were retained for the multivariable analysis. The null hypothesis (H0) aimed to prove the independence between the infection status and the corresponding risk factors (categorical variables). Data entry, cleaning, and pivoting was performed in Microsoft EXCEL. Statistical modeling was done in R Studio 2021.09.0 [(30), http://www.rstudio.com/]. Spatial data visualization was done in QGIS 3.26 (QGIS Geographic Information System 2022 http://www.qgis.org).

## Results

### Individual parameters and seroprevalence

By ELISA, 4.0% of samples (31/780) showed antibodies against *N. caninum*. Inconclusive results were recorded in 0.6% of samples (5/780). These samples were tested by immunoblot, which yielded two further positive results, making the final apparent seroprevalence 4.2% (33/780, 95% CI: 2.8–5.6%). Most of the sampled cattle were aged between 3 and 6 years. Only one seropositive animal was < 1 year old and another seropositive animal was 1 year old. The 31 remaining positive cattle were adults ranging from 2 to 15 years of age (Table 1). The cattle corresponded to 16 different cattle breeds excluding crossbreeds. Most sampled cattle belonged to the breed brown cattle (Braunvieh) 30.6% (239/780). Red Holstein and Holstein were sampled in equal proportions (15%), while the other types of breeds were present in a small percentage (Table 2). Seropositive animals were observed among nine of those breeds: Brown cattle (*n* = 7), Red Holstein (*n* = 7), Swiss Fleckvieh (*n* = 6), Red Fleckvieh (*n* = 4), Holstein (*n* = 3), Gray cattle (*n* = 2), Normande (*n* = 2), Original Brown Swiss (*n* = 1), and Simmental (*n* = 1) (Table 2).

**Table 1:** Age group structure of sampled female cattle (*n* = 780) and serological status for *N. caninum*, ^*^only age group is known (i.e., adults), date of birth was not recorded.

**Age group**	**Years of age**	***n* positive**	**% positive**	***n***
Calves (<1 year old)	0	1	20.0	5
Yearlings (1 ≤ years old < 2)	1	1	10.0	10
Adults (≥2 years old)	2	3	3.9	77
	3	3	2.0	154
	4	8	6.5	124
	5	1	0.8	119
	6	6	6.7	89
	7	1	1.6	61
	8	1	2.0	49
	9	2	7.4	27
	10	2	9.1	22
	11	2	20.0	10
	12	0	0.0	6
	13	0	0.0	4
	14	1	50.0	2
	15	1	33.3	3
	16	0	0.0	1
	na*	0	0.0	17
Total		33	4.2	780

**Table 2:** Breed composition in the sampled female cattle and seroprevalence according to breed, na^*^ breed not recorded.

**Breed**	**% breed**	***n* total**	**% positive**	***n* positive**
Brown cattle	30.6	239	2.9	7
Red Holstein	15.8	123	5.7	7
Holstein	15.5	121	2.5	3
Swiss Fleckvieh	9.1	71	8.5	6
Simmental	6.7	52	1.9	1
Red Fleckvieh	5.4	42	9.5	4
Original Brown Swiss	3.2	25	4.0	1
Montbéliard	2.8	22	0.0	0
Gray cattle	2.4	19	10.5	2
Jersey	1.7	13	0.00	0
Normande	0.9	7	28.6	2
Limousin	0.6	5	0.00	0
Eringer	0.5	4	0.00	0
Évolène	0.4	3	0.00	0
Highland	0.4	3	0.00	0
Hinterwälder	0.1	1	0.00	0
Crossbreed	2.3	18	0.00	0
na*	1.5	12	0.00	0
Total	100.0	780	4.2	33

### Farm parameters and seroprevalence

**Figure 1:**
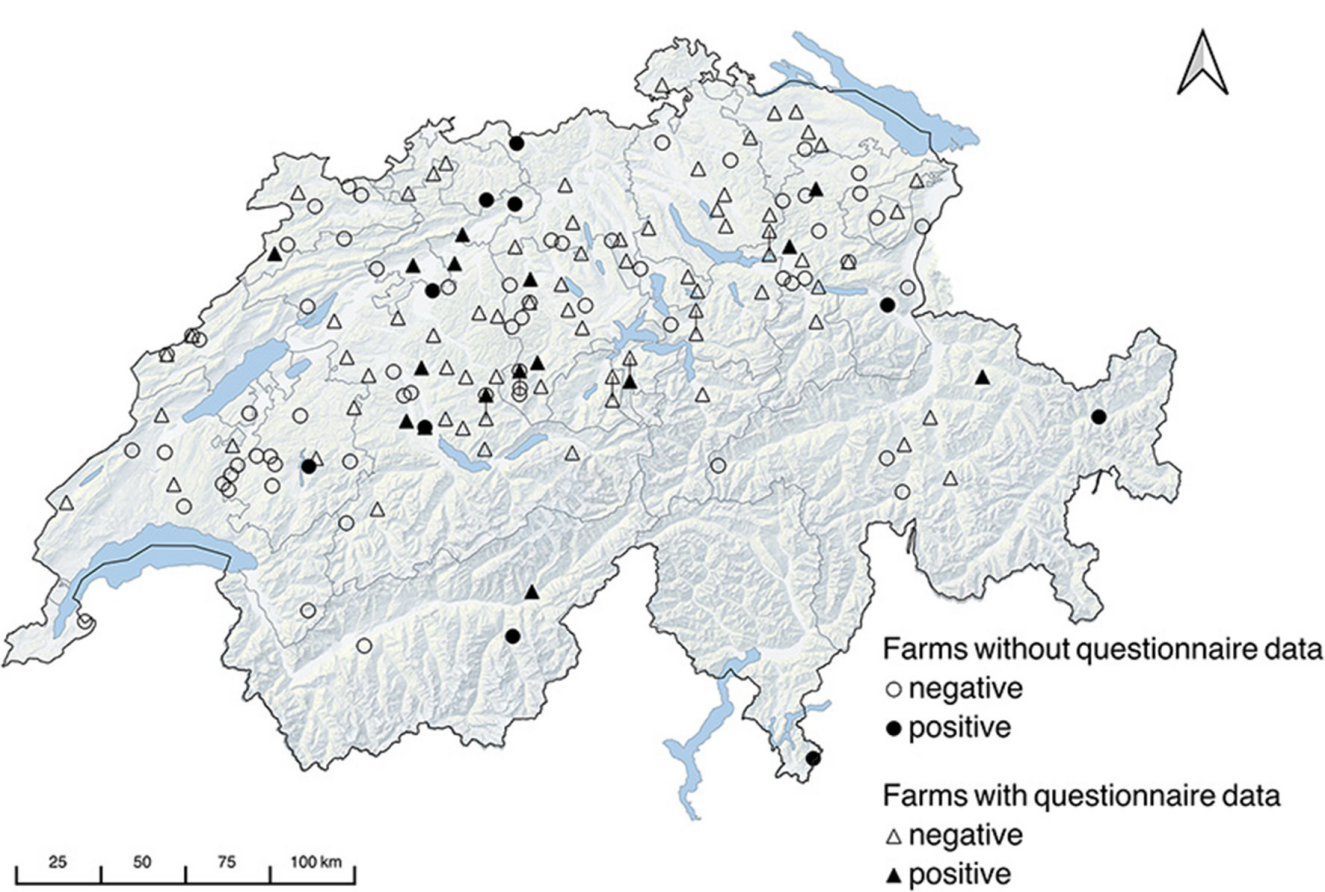
Map of Switzerland with geographical distribution of the sampled farms plotted according to the zip code. Locations with identical zip codes are displaced by bars.

The response rate of valid questionnaires was 53.4% (87/161). Almost half of the farms were dairy farms (54.0%, 47/87). Purely beef producing farms were 8.0% (7/87), and 31.0% (27/87) of the farms had mixed production with dairy and beef cattle. Six farms were commercially rearing heifers as sole production type (4.6%, 4/87) or besides milk or beef production (2.2%, 2/87). The main way of restocking was rearing of replacement heifers (58.6%, 51/87), followed by a mixed restocking through rearing of replacement heifers and buying-in (23.0%, 20/87). Most herds had access to pasture in the area (55.2%, 48/87) and most farms sent at least a part of their herd for summer grazing to Alpine pastures (60.9%, 53/87). In less than half of the farms (40.2%, 35/87), pasture represented 25 to 50% of the whole feed ration. In 36.8 % (32/87) of the farms the proportion of pasture to whole feed surpassed 50%. Feed storage was done in 60.9% (53/87) of cases in closed compartments inaccessible to dogs (e.g., silos, hayloft). The majority of farmers (69.0%, 60/87) reported abortions in cattle in the previous 3 years and 35.0% (21/60) of these submitted samples for diagnosis. Only one case of abortion was confirmed to have been caused by *N. caninum*. More than half of the farms (56.3%, 49/87) reported owning dogs. Almost all dog-owning farmers fed their dog canned or dry dog food (95.9%; 47/49) and 42.9% (21/49) also supplemented with leftovers. Only in one case the dog was fed leftovers alone and in two cases raw meat was added. Five owners reported that their dogs fed on hunted mice or small birds. Farm dogs or dogs from strangers had access to the pastures in all farms, and in 43.7% of farms (38/87) they could even access the stables. Rodents were reported to be a problem in one third of the farms (29.9%, 26/87). Most farms (83.9%, 73/87) housed at least one other animal species: cats (43.7%, 38/87), chicken (43.7%, 38/87), sheep (16.1%, 14/87), goats (25.3%, 22/87) and <12.6% other species, namely horses, donkeys, rabbits, pigs, or waterfowl, respectively. A farm was considered positive if it had at least one animal tested positive; thus, 16.1% (26/161) of the sampled farms were positive. These farms originated from 12 of the 25 sampled cantons. The geographical distribution of the sampled farms indicating the serological results is shown in Figure 1. Questionnaire data were available for only 16 of the 26 positive farms. Of the 16 farms, six were dairy farms, five were mixed production dairy-beef, three were beef farms, one was heifer rearing, and one was mixed dairy-beef-heifer rearing. Regarding herd size, 18.8% (3/16) were large (more than 32 animals), 37.5% (6/16) were medium-sized (23 to 32 animals), and 43.6% (7/16) were small (fewer than 23 animals). The restocking in the seropositive farms was done almost in equal proportion for each category: rearing of replacement heifers 31.3% (5/16), buying-in 31.3% (5/16), mix of both 37.5% (6/16). Only two of the seropositive farms did not record pasture access near stable, and these also did not perform alpine grazing, unlike the rest. Fourteen of the 16 seropositive farms provided more than 25% pasture in relation to whole feed, six of which provided more than 50%. Feed was closed in nine farms vs. open in seven farms. Twelve of the seropositive farms reported cattle abortions in the previous 3 years. Dog ownership and dog access to stable was reported in nine seropositive farms. Problems with rodents were reported in half of the seropositive farms.

### Risk factors

The tested variables are displayed in Table 3. In a first attempt, the effect of each explanatory variable was tested independently on the farm serostatus or animal serostatus. Variables that were highly heterogenous were pooled depending on their biological relevance. The count of dogs with access to stable were summed from farm-owned dogs and visiting from neighbor or tourists. The most commonly “other animal species” housed in the farms were cats, chickens, sheep, and goats. The count of the last two species were merged as “small ruminants.” Other species were not considered in the risk analysis due to low numbers. At the animal level “rodents are a problem” was the only significant factor for *N. caninum* seropositivity (OR: 3.77, 95%CI: 1.029–13.77, *p* = 0.045).

**Table 3:** Summary of farming parameters based on answers in the questionnaire and farm serostatus.

**Farm variable**	**Variable level**		**Count of seropositive farms**	**% seropositive farms**	***n***
Type of production	Rearing		1	25.0	4
	Beef		3	42.9	7
	Dairy		6	12.8	47
	Dairy and rearing		0	0.0	1
	Dairy and beef		5	18.5	27
	Dairy and rearing and beef		1	100.0	1
Restocking	Rearing of replacement heifers + Buying-in		6	30.0	20
	Rearing of replacement heifers		5	9.8	51
	Buying-in		5	33.3	15
	na		0	0.0	1
Herd size	Large (>32 animals)		3	10.3	29
	Medium (23–32 animals)		6	21.4	28
	Small (<23 animals)		7	23.3	30
Housing	Stable + pen		2	28.6	7
	Stable + pen + pasture		7	14.6	48
	Stable + pasture		7	21.9	32
Alpine summer grazing	Yes		5	16.1	31
	No		3	10.0	30
	Yes, a part of the herd		6	27.3	22
	na		2	50.0	4
Water source	Ground water		8	21.6	37
	Ground water + pond		0	0.0	1
	Tap water		7	17.5	40
	Tap + ground water		0	0.0	8
	Tap + ground water + pond		1	100.0	1
Feeding	Pasture/cut grass	No	1	50.0	2
		Yes	15	17.7	85
	Hay	No	1	100.0	1
		Yes	15	17.4	86
	Grass silage	No	6	23.1	26
		Yes	10	16.4	61
	Corn silage	No	10	24.4	41
		Yes	6	13.0	46
	Concentrated feed	No	7	36.8	19
		Yes	9	13.2	68
% pasture in whole feed	< 25%		2	10.5	19
	>50%		6	18.8	32
	25–50%		8	22.9	35
Feed storage	Closed		9	17.0	53
	Open		7	20.6	34
History of abortion	No		3	13.6	22
	Yes		12	20.0	60
	Don't know		1	20.0	5
If yes, how many	< 5		8	22.9	35
	5 or more		3	18.8	16
	Don't know		1	11.1	9
If yes, diagnostics performed	No		9	23.1	39
	Yes		3	14.3	21
If diagnostics yes,	*N. caninum*		0	0.0	1
what was the cause	Not *N. caninum*		0	0.0	1
	Don't know		0	0.0	1
Dog ownership	No		7	18.4	38
	Yes		9	18.4	49
Dogs access to stable	No		7	15.9	44
	Yes		9	20.9	43
Dog food	Canned/dry dog food				
		No	7	17.5	40
		Yes	9	19.2	47
	Leftovers				
		No	1	100.0	1
		Yes	15	17.4	86
	Raw meat				
		No	6	23.1	26
		Yes	10	16.4	61
	Dog hunts mice/birds				
		No	16	19.5	82
		Yes	0	0.0	5
Problems with rodents	No		8	13.1	61
	Yes		8	30.8	26
Other animal species present	No		5	35.7	14
	Yes		11	15.1	73

Some variables were pooled because of low counts.

At farm level, two significant protective factors for seropositivity were identified, namely restocking by self-rearing (OR: 0.25, 95%CI:0.06–0.96, *p* = 0.043) and feeding concentrated feed (OR: 0.26, 95%CI: 0.08–0.86, *p* = 0.024). These two factors were no longer significant when they were tested together in the multivariable model (Table 4).

**Table 4:** Models that rendered a *p*-value ≤ 0.1 (“ref” reference, “OR” odds ratio, “LL” and “UL” lower limit and upper limit of 95% confidence interval).

**Response variable**	**Explanatory variable**	**Variable level**	**OR**	**LL**	**UL**	***z*-value**	***p*-value**
Individual serostatus (animal level)	Age	Intercept	0.006	0.001	0.031	−6.000	< 0.001
		Age in years	1.130	0.985	1.295	1.745	0.081
	Feeding of concentrated feed	Intercept	0.045	0.010	0.202	−4.065	< 0.001
		No (ref)					
		Yes	0.242	0.055	1.057	−1.886	0.059
	Rodent problem	Intercept	0.012	0.003	0.049	−6.032	< 0.001
		No (ref)					
		Yes	3.765	1.029	13.773	2.003	0.045
Farm Serostatus (farm level)	Restocking	Intercept	0.429	0.152	1.068	−1.736	0.083
		Rearing of replacement heifers + Buying-in					
		Rearing of replacement heifers	0.254	0.064	0.960	−2.023	0.043
		Buying-in	1.167	0.268	4.977	0.210	0.834
	Feeding of concentrated feed	Intercept	0.583	0.217	1.450	−1.133	0.257
		No (ref)					
		Yes	0.262	0.081	0.855	−2.254	0.024
	Rodent problem	Intercept	0.151	0.066	0.299	−4.985	< 0.001
		No (ref)					
		Yes	2.944	0.956	9.165	1.896	0.058
	Other animals species	Intercept	0.556	0.171	1.608	−1.054	0.292
		No (ref)					
		Yes	0.319	0.091	1.199	−1.765	0.078

## Discussion

Abortions are a clear issue in cattle husbandry as almost three quarter of the questioned farms had experienced abortions in the previous 3 years. Additionally, some abortions may go undetected, especially during summer grazing. This is a traditional practice in Switzerland that takes place from mid-June to end of August (31). Abortions or stillbirths that take place during the grazing season may be missed due to the wide movement range of the cattle, especially on Alpine pastures, and the removal of any remnants by scavenging animals. In Switzerland, *N. caninum* has been recognized as the most important infectious abortion cause in cattle (22, 23, 32). In line with this, a retrospective study on abortion related samples submitted for diagnostic from 2011 to 2019 to the Institute of Parasitology Bern detected *N. caninum* DNA by Real Time PCR in brain samples from 16.6% (165/992) of the analyzed bovine fetuses (33). Only one representative study on the seroprevalence of *N. caninum* in Swiss cattle had been done over 20 years prior to our study and found a 11.5% seroprevalence (22), which is 2.7-fold higher than in the present study. Some factors could have led to this decline: bovine neosporosis is a reportable disease since 2001, and while *N. caninum* diagnostic is not included in the mandatory panel of investigations of repeated abortions, financial support for diagnostic approaches may be available on the farmers' request in some cantons. This can lead to an early identification of positive cases in a farm and early implementation of measures. Also, awareness of farmers toward this parasitic abortive agent may have increased over the last decades, aided by its status as reportable disease. The low overall prevalence could be explained also by the endemicity of *N. caninum* infections. At farm level *N. caninum* can cause abortion storms (34) and remain a persistent abortifacient pathogen. Cattle derived from more than 30 farms in Switzerland that have experienced repeated abortions from 2010 through 2022 tested seropositive by ELISA (unpublished data) at the Institute of Parasitology in Bern, Switzerland, suggesting *N. caninum* as a possible cause of abortion. By multilevel modeling we observed a 3.7 times odds of animal seropositivity when farmers reported to have “a problem with rodents” at the farm. Another study identified exposure to rodents as a risk for seropositivity but with a 1.7 times odds (35). How rodents might be linked to serostatus of the cattle remains to be investigated, but one possibility is that they act as intermediate hosts that may transmit the parasite to canids (6). In our study, we identified restocking by rearing of replacement heifers as a protective factor. This could be explained, as new animals from different origins may introduce *N. caninum* unnoticed into the farm and transmit it further vertically to new generations. This finding also supports the recommendation to test new breeding animals for antibodies against *N. caninum* prior to bringing them to the herd. Cattle farming in Switzerland appears to be shifting toward greater herd size as farms with >30 animals are on the rise (36). This could pose further challenges when dealing with neosporosis, as to this date testing of individual animals is recommended to reliably detect infected cows. So far, testing of pooled serum samples by commercial kits did not prove sensitive enough, most likely due to high variation of individual antibody titers according to the gestation progress (37). If this could be overcome, it could become a cost-effective way to assess *N. caninum* serostatus at herd level. Another possibility is to test bulk milk for antibodies by ELISA (38), an attractive approach for dairy herds. Interestingly, already reported risk factors in other settings such as presence of dogs in farm (39) were not observed in the present study. Although we considered the sample size adequate, the overall prevalence may be too low to infer more significant risk factors. A future reassessment of the bovine neosporosis seroprevalence should consider the herein reported seroprevalence and adjust the sample size accordingly. Although the response rate of the questionnaire (53.4%, *n* = 161) was acceptable when compared to other survey studies on cattle farming practices: 27%, *n* = 1,974 (40) and 28.3%, *n* = 1,835 (41), a clear limitation in the risk factor analysis was posed by the highly heterogeneous answers. In addition, the interpretation of the free answers (as most could not be categorized) and the incomplete filling of the form decreased the power of the analysis. This could be improved in future by reducing the questionnaire complexity. Furthermore, we cannot exclude recall bias because some questions had a retrospective nature. The participating farm will be provided with a brief report of the outcome of the study, where the importance of their participation will be stressed. Hopefully this will increase their interest in participating in future studies that will have to show whether more risk factors apply to the specific situation in Switzerland. Measures of prevention should focus on improving farm management and diagnostics. With reliable serological test at herd levels still missing, control measures still rely on individual serological testing of breeding animals, together with breeding exclusion of seropositive mothers. Crossbreeding with beef bulls has been shown to reduce the risk of abortion in seropositive dairy cows (42). Our study reports an apparent drop in seroprevalence for *N. caninum* in the Swiss cattle population over the last decades, and we argue that this is thanks to the status as a reportable disease of bovine neosporosis. Continuous effort should be made to screen for *N. caninum* in herds with repeated reproduction losses, identify the mode of transmission and implement case-specific measures of control and prevention.

## Data availability statement

The original contributions presented in the study are included in the article (Supplementary material 2), further inquiries can be directed to the corresponding author/s.

## Ethics statement

The animal study was reviewed and approved by the Swiss Cantonal Committees for animal experimentation in each canton (approval number BE 5/17+). Written informed consent was obtained from the owners for the participation of their animals in this study.

## Author contributions

DG conducted laboratory work, data curation, statistical analysis, and wrote the first draft. WB was involved in study planning, supervision of laboratory work, result interpretation, and critical review of the manuscript. FA and GM-J collected the samples. GS performed statistical analysis and revised the manuscript critically. PZ was involved in conceptualization of the study, study planning, sample collection, and data curation. CF was involved in conceptualization of study, analysis, and writing. All authors read and approved the final manuscript.

## Funding

The Federal Food Safety and Veterinary Office in Switzerland covered the costs of the test kits for serology.

## Conflict of interest

The authors declare that the research was conducted in the absence of any commercial or financial relationships that could be construed as a potential conflict of interest.

## Publisher's note

All claims expressed in this article are solely those of the authors and do not necessarily represent those of their affiliated organizations, or those of the publisher, the editors and the reviewers. Any product that may be evaluated in this article, or claim that may be made by its manufacturer, is not guaranteed or endorsed by the publisher.
