# Supplementary material for: Switzerland-wide Neospora caninum seroprevalence in female cattle and identification of risk factors for infection
Source: Front Vet Sci. 2022 Nov 17;9:1059697. doi: 10.3389/fvets.2022.1059697 (PMC9714321; doi:10.3389/fvets.2022.1059697)
Supplement: Supplementary file 1 [file Data_Sheet_1.docx]

Switzerland-wide *Neospora caninum* seroprevalence in female cattle and identification of risk factors for infection

Diana S. Gliga^1*^, Walter Basso^1^, Flurin Ardüser^2,4^, Gaia Moore-Jones^2,5^, Gereon Schares^3^, Patrik Zanolari^2†^, Caroline F. Frey^1^

^1^Institute of Parasitology, Department of Infectious Diseases and Pathobiology, Vetsuisse Faculty, University of Bern, Bern, Switzerland

^2^Clinic for Ruminants, Vetsuisse Faculty, University of Bern, Bern, Switzerland

^3^Institute of Epidemiology, Friedrich-Loeffler-Institut, Federal Research Institute for Animal Health, Greifswald-Insel Riems, Germany

^4^Current affiliation: Gross- und Kleintierpraxis Lai, Lenzerheide, Switzerland

^5^Current affiliation: Amt für Veterinärwesen, Bern, Switzerland

*****Corresponding Author
diana.gliga@unibe.ch

Questionnaire survey for cattle farms in Switzerland

# 1. General information about owner and farm

## Owner: name, address, telephone number, e-mail

Farm: registration number; production type (dairy, beef, dairy+beef, other: …….)

Number of animals

Restocking: rearing of replacement heifers (yes/no), buying-in (yes/no), rearing of replacement heifers + buying-in (yes/no)

# 2. Information on bovine husbandry and bovine health

Type of husbandry:

stable and pen: tie-stall (yes/no), free stall (yes/no)

stable and local pasture: tie-stall (yes/no), free stall (yes/no)

alpine summer grazing (yes/no)

Type of water supply:

public supply water (yes/no)

private water source: groundwater (by water pump) or spring (yes/no)

pond/stream (yes/no)

other: …….

Feed for cattle:

pasture/ fresh grass (yes/no), hay (yes/no), grass silage (yes/no), corn silage (yes/no),

concentrated feed (yes/no), other: …….

Proportion of pasture in whole feed (if grazing):

<25%, 25 - 50%, or >50%

Storage of feed:

open (dog has access) (yes/no); closed (yes/no), how is it closed: …….

History of cattle abortion in the previous 5 years?

don't know

no

yes, how many: …….

- - if yes, was aborted material examined (yes/no)
    - if yes, were abortifacient pathogens detected? (yes/no)
      - if yes, which ones: *Neospora caninum* (yes/no), others: …….

# 3. Information on dog ownership

Have there been own dogs on the farm in the last 6 years? If so, tick affected years.

no

yes: 2021, 2020, 2019, 2018, 2017, 2016, 2015

- If yes:

Did your own farm dogs have access to: stable (yes/no), pasture (yes/no), feed storage (yes/no)?

Have there been puppies (≤6 months) on the farm in the last 4 years? (yes/no/don’t know)

Do foreign dogs (e.g., dogs of hikers) have access to: stable (yes/no), pasture (yes/no), feed storage (yes/no)?

What are the dogs fed with? canned/dry food (yes/no), leftovers (yes/no), raw meat (yes/no), dog hunts rodents/birds (yes/no)

# 4. Information on other animal species

Have you had problems with rodents (mice, rats) in the last 2 years? (yes/no)

Do you keep other animal species besides cattle (and dogs) on the farm? If so, which animal species and how many animals each?
